# Investigating emotional contagion in dogs (*Canis familiaris*) to emotional sounds of humans and conspecifics

**DOI:** 10.1007/s10071-017-1092-8

**Published:** 2017-04-21

**Authors:** Annika Huber, Anjuli L. A. Barber, Tamás Faragó, Corsin A. Müller, Ludwig Huber

**Affiliations:** 1 Clever Dog Lab, Comparative Cognition, Messerli Research Institute, University of Veterinary Medicine Vienna, Medical University of Vienna, University of Vienna, 1210 Vienna, Austria; 2 MTA-ELTE Comparative Ethology Research Group, Pázmány Péter sétány 1/C, 1117 Budapest, Hungary

**Keywords:** Empathy, Emotional contagion, Playback study, Dogs

## Abstract

Emotional contagion, a basic component of empathy defined as emotional state-matching between individuals, has previously been shown in dogs even upon solely hearing negative emotional sounds of humans or conspecifics. The current investigation further sheds light on this phenomenon by directly contrasting emotional sounds of both species (humans and dogs) as well as opposed valences (positive and negative) to gain insights into intra- and interspecies empathy as well as differences between positively and negatively valenced sounds. Different types of sounds were played back to measure the influence of three dimensions on the dogs’ behavioural response. We found that dogs behaved differently after hearing non-emotional sounds of their environment compared to emotional sounds of humans and conspecifics (“*Emotionality*” dimension), but the subjects responded similarly to human and conspecific sounds (“*Species*” dimension). However, dogs expressed more freezing behaviour after conspecific sounds, independent of the valence. Comparing positively with negatively valenced sounds of both species (“*Valence*” dimension), we found that, independent of the species from which the sound originated, dogs expressed more behavioural indicators for arousal and negatively valenced states after hearing negative emotional sounds. This response pattern indicates emotional state-matching or emotional contagion for negative sounds of humans and conspecifics. It furthermore indicates that dogs recognized the different valences of the emotional sounds, which is a promising finding for future studies on empathy for positive emotional states in dogs.

## Introduction

Empathy is a social phenomenon that can be defined as “the capacity to […] be affected by and share the emotional state of another” (de Waal 2008, p. 281). Preston and de Waal (2002) proposed a multi-level concept of empathy, whose most basic level is emotional contagion, defined as an automatic and unconscious emotional state-matching between two individuals (Preston and de Waal 2002; de Waal 2008). Hatfield et al. (1993) considered the functional significance of emotional contagion as: “[…] [It] may well be important in personal relationships because it fosters behavioural synchrony and the moment-to-moment tracking of other people’s feelings even when individuals are not explicitly attending to this information” (Hatfield et al. 1993, p. 96). Consequently, emotional contagion can have important facilitation effects in the social domain, which is not only restricted to our own species’ social life—emotional contagion is a phylogenetically old capacity (Palagi et al. 2014), which suggests the existence of this emotional level of empathy also in non-human animals.

Emotional contagion has been demonstrated in various animal species ranging from primates (e.g. Parr 2001; Ross et al. 2008; Palagi et al. 2009, 2014) to rodents (e.g. Langford et al. 2006; Bartal et al. 2011, 2014) and avian species (e.g. Edgar et al. 2011; Osvath and Sima 2014), and it has been suggested in farm animals (Reimert et al. 2013, 2015). Furthermore, evidence for emotional contagion has been found in pet animals, more precisely in dogs (e.g. Zahn-Waxler et al. 1984; Joly-Mascheroni et al. 2008; Custance and Mayer 2012; Silva et al. 2012; Madsen and Persson 2013; Yong and Ruffman 2014; Palagi et al. 2015; but see Harr et al. 2009; O’Hara and Reeve 2011). Dogs are exceptional as several studies have indicated that they express empathy even towards other species, in this case humans (e.g. Zahn-Waxler et al. 1984; Custance and Mayer 2012; Sümegi et al. 2014; Yong and Ruffman 2014). This cross-species occurrence of empathy makes further research with dogs highly valuable as a resource for increasing our understanding of this social phenomenon (see for an opinion Silva and de Sousa 2011).

Empirical evidence about dogs’ sensitivity to emotional states in humans is accumulating. Recently, a study has demonstrated dogs are capable of discriminating between different emotional expressions in human faces (Müller et al. 2015); similar results on heterospecific emotion discrimination have also been found in horses (Smith et al. 2016a; but see Schmoll 2016; Smith et al. 2016b). Furthermore, on a multimodal level combining visual with acoustic stimuli, dogs can extract, integrate, and discriminate humans’ as well as conspecifics’ emotional expressions (Albuquerque et al. 2016). Focussing particularly on empathy, a recent questionnaire study investigated dog owners’ evaluation of empathic-like responding in their dogs (Szánthó et al. 2017). However, apart from this first survey-based approach of the topic, previous studies focussed on behavioural observations of dogs confronted with emotional situations in an intraspecies context and an interspecies context. Thus, dogs have been shown to express comfort-offering behaviours to a human suddenly pretending to cry—a response that has been interpreted as demonstrating empathic-like behaviour driven by emotional contagion (Custance and Mayer 2012). This does not, however, imply that dogs understand the nature of the cry as an expression of emergency or the need to obtain help for the crying person. In a study by Macpherson and Roberts (2006), dogs failed to solicit help from a bystander when the dog’s owner feigned a heart attack or experienced an accident. Still, this is not evidence that dogs are apathic towards people in need and thus not evidence against empathic-like behaviour driven by emotional contagion.

Given dogs’ sensitivity to vocal emotional valence features of human and conspecific sounds (Andics et al. 2014), it is likely that an empathetic response is induced in dogs by solely hearing an emotional vocalization. Accordingly, on an interspecies level, a playback study that investigated dogs’ responses to vocalizations of human distress, specifically human infant crying, provided evidence for emotional contagion in dogs triggered solely by the detection of the heterospecifics’ acoustic cues (Yong and Ruffman 2014). Finally, another playback study found evidence for an empathetic response to distress vocalizations in dogs on an intraspecies level (Quervel-Chaumette et al. 2016). Thus, both an interspecies empathetic response and an intraspecies empathetic response to negatively valenced emotional states, triggered solely by acoustic stimuli, could be demonstrated in dogs.

Our aim was to further shed light on emotional contagion in dogs to acoustic stimuli of different meanings. Therefore, we applied a playback study where we analysed for the first time three dimensions simultaneously: first, we analysed the “*Emotionality*” dimension by contrasting non-emotional sounds of the dogs’ environment with emotional sounds of humans and conspecifics. Second, we analysed the “*Species*” dimension by contrasting emotional sounds of humans with those of conspecifics. Third, we analysed the “*Valence*” dimension by contrasting positively with negatively valenced sounds of both species. Comparing these different dimensions within one experimental paradigm provides the possibility to simultaneously gain new insights into intra- and interspecies empathy in dogs as well as into differences in responses relating to positive and negative emotional states. This is important both from a fundamental science perspective to increase our knowledge on animal emotions and behaviour as well as for considering possible additional effects of domestication and socialization in terms of canine emotional contagion. Along with the functional significance of emotional contagion (see Hatfield et al. 1993), one could expect that the human–dog relationship can also be facilitated by heterospecific emotional contagion. Thus, research on this phenomenon could provide new insights concerning influential but so far less considered effects on this interspecies relationship. Apart from that, knowledge on canine emotional contagion is important from an applied perspective in terms of relevant effects on animal welfare, i.e. when dogs emotionally resonate with vocalizations they hear from humans and other dogs, and this will affect their own emotional state and, consequently, their welfare.

We evaluated the dogs’ behavioural responses towards their owner and towards a loudspeaker broadcasting the stimulus. Furthermore, we analysed behaviours that have been identified in previous studies as being indicative for arousal and negative emotional states in dogs. For the “*Emotionality”* dimension, we hypothesized dogs perceive the emotional features of sounds, based on findings that they are sensitive to emotional sounds of humans and conspecifics (Andics et al. 2014) and behave in expected manner to them (Custance and Mayer 2012; Yong and Ruffman 2014; Quervel-Chaumette et al. 2016). Therefore, we predicted the dogs’ behavioural response would differ between emotional and non-emotional sounds. Specifically, we predicted the subjects would express an increased attentiveness to emotional sounds represented by an increased duration of orientation towards the loudspeaker they heard the stimulus from and a reduced orientation towards their owner. Furthermore, the behavioural indicators for arousal and negative emotional states should be increased in response to emotional sounds. For the “*Species”* dimension, we hypothesized dogs express comparable empathetic responses in both an intraspecies context and interspecies context due to their sensitivity to emotional sounds of both humans and conspecifics (Andics et al. 2014) and due to previous studies demonstrating an emotional contagion response to emotional vocalizations of both (Yong and Ruffman 2014; Quervel-Chaumette et al. 2016). If this is true, then we should not find a difference in the behavioural reaction towards emotional human and conspecific sounds. For a reasonable interpretation of the dogs’ response being triggered by emotional contagion, the “*Valence”* dimension is, besides the premise that the subjects recognize emotional features of sounds (“*Emotionality”* dimension), decisive. Based on findings of previous studies (e.g. Yong and Ruffman 2014; Quervel-Chaumette et al. 2016), we hypothesized dogs demonstrate emotional contagion at least in response to negative emotional sounds of both species. As emotional contagion implies an emotional state-matching with the other (de Waal 2008), dogs should express behavioural indicators for emotional states that match the valence of the sound stimulus. Consequently, we predicted that dogs’ behavioural response should differ between positively and negatively valenced stimuli. Specifically, the analysed indicators for arousal and negative emotional states have to be significantly increased in response to negative emotional sounds compared to positive ones.

Distinctively demonstrating an emotional contagion response to positive emotional sounds requires evidence that behavioural indicators referring to positive emotional states are increasingly expressed. However, compared to our knowledge about negative emotions in dogs, which is already relatively advanced, we still lack comprehensive and validated behavioural indicators for the positive counterpart that could feasibly be analysed in this study. Therefore, we focussed on investigating whether indicators for negative emotional states were expressed significantly less in response to positive sounds. If so, and if this is the only difference in the dogs’ response to both types of emotional sounds, it would indicate that the subjects recognized the different valences, which is an essential first step towards future studies investigating emotional contagion for positive valences as well.

## Materials and methods

### Subjects

We recruited 53 adult pet dogs (33 females, 20 males, *mean age* = 4.87 years, *SD* = 3.01) of different breeds as well as mongrels (see appendix for detailed information, Table 6) with private owners (45 women, 8 men). The minimum age of the dogs was 1 year. Dogs were recruited from the Clever Dog Lab database, from local canine clubs, and with flyers distributed online. One of the participating dogs dropped out of the study after the first session as the owner did not come for the second session. Due to technical problems, one subject heard one playback less often than the others.

### Ethical approval

All procedures applied in this study were discussed and approved by the institutional ethics committee of the University of Veterinary Medicine Vienna in accordance with good scientific practice guidelines and national legislation (Ref. 06/04/97/2014). All dog owners volunteered to participate with their dogs in this study and gave written consent. The experimental procedure was purely non-invasive, and handling of the dogs was always in a positive and pleasant manner.

### Apparatus and set-up

**Fig. 1:**
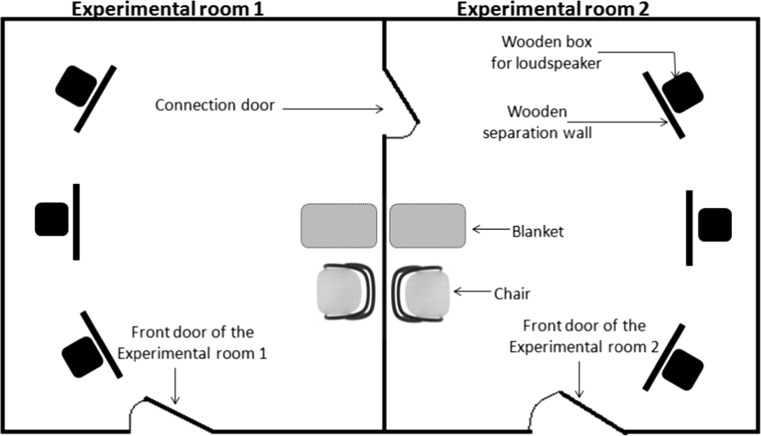
Schematic sketch of the experimental rooms (1 and 2) with the required objects (three wooden separation walls and boxes per room to hide the loudspeaker, one blanket, and one chair per room)

Testing took place in two equally sized (6 m × 3 m) and identical experimental rooms at the Clever Dog Lab (University of Veterinary Medicine Vienna). These rooms were connected by a door. In each room, three wooden separation walls were arranged in a semicircular manner (Fig. 1). Behind each separation wall, there was a wooden box, which served as a hiding place for the external loudspeaker (Technaxx MusicMan Mini, frequency range 150–18,000 Hz ± 3 dB) from which the audio stimuli were played back. There was one loudspeaker per room, which was connected via an audio cable to a laptop outside. The speaker was shifted by the experimenter between the three wooden boxes of each room randomly for each trial, which was part of the procedure to change the situational context for each trial to hinder habituation (see also “Procedure” section). At the opposite wall, and in line with the central separation wall, a blanket for the dog was put on the ground. For the dog owner, a chair was placed beside the dog’s blanket.

### Stimuli

For the “*Species”* dimension, acoustic stimuli of both humans and conspecifics were played back. For the “*Valence”* dimension, stimuli covered positively and negatively valenced sounds of both species. For the “*Emotionality”* dimension, sounds of humans and conspecifics with both valence types (positive and negative) were pooled as emotional sounds. Non-emotional stimuli contained both biotic (from living organisms) and abiotic (non-living elements) stimuli from the dogs’ natural environment.

For the emotional stimuli of humans, laughing was played back as a positive and crying as a negative stimulus. For the emotional stimuli of dogs, play barks were used as a positive and isolation whines as a negative stimulus. Non-emotional stimuli consisted of five different sound types, from which four sounds were selected for each subject, containing both abiotic and biotic sounds. Abiotic sounds contained the sound of rain and leaves rustling in the wind. Human female neutral talking, blackbird singing (*Turdus merula*), and the sound of a cricket (*Tettigonia cantans*) were summarized under biotic sounds.

Human stimuli were obtained from the Montreal Affective Voices database (http://vnl.psy.gla.ac.uk) and from a public sharing website (http://www.youtube.com). Dog stimuli were from the dog vocalization database of the Department of Ethology, Budapest (available upon request from TF). The isolation whines were recorded in the laboratory when a dog was separated from the owner (during the experiment published in Konok et al. 2011). The play barks were recorded by Csaba Molnár for studies on barking (e.g. Pongrácz et al. 2005). Non-emotional stimuli were obtained from online databases (http://www.tierstimmenarchiv.de, http://www.youtube.com, http://www.freesound.org).

All recordings were edited using Audacity software (Audacity version 2.0.3). The frequency of the stimuli was not manipulated to avoid the loss of context and valence of the sounds. From each sound clip, 5 s were cut out and a stimulus was created comprising 5 s playback—10 s silence—5 s playback. For each stimulus type, we prepared different versions (three for each emotional sound type, two for each biotic and one for each abiotic non-emotional sound type) that were used alternately. Each stimulus was played back at a natural volume with loudness naturally oscillating around a mean value ranging between 60 and 70 dB.

### Procedure

The experiment consisted of two sessions with four trials each. Thus, in each session, four different acoustic stimuli were played back, corresponding to the following rules we considered more appropriate than a fully balanced design: (1) the first and the last stimulus were always emotional, whereas the second and third stimulus were always non-emotional sounds (“*Emotionality”* dimension). The reason for this rule was to provide the maximum time between the emotional stimuli to avoid after-effects of the first emotional stimulus being present during the second one. (2) In each session, both an emotional human and an emotional dog sounds were played back (“*Species”* dimension). We randomly assigned to each subject which of these sounds were played back in the first and in the last trial, respectively. If the first trial of the first session was an emotional human sound, the first trial of the second session was an emotional dog sound and vice versa. (3) The emotional valence of these stimuli was opposed; thus, when the emotional human sound had a positive valence, the emotional dog sound had a negative one and vice versa (“*Valence”* dimension). In the second session, the respective valence of human and dog sounds was switched, and thus, when the human sound had a positive valence in the first session, it had a negative one in the second, likewise for the dog stimuli. According to these rules, we had four different stimulus set combinations (I–IV in Table 1) differing in whether an emotional dog or an emotional human sound was played back in the first, respectively, fourth trial as well as which of them was positively or negatively valenced. For the non-emotional stimuli, we played back three biotic and one abiotic sound to each subject. We prepared 24 stimulus sets which always corresponded to the predefined rules (see Table 1), but contained different versions of the relevant stimuli. The stimulus set presented was randomly assigned to each subject.

**Table 1:** Overview of possible stimulus set combinations used in the present study

Possible stimulus set combinations	Session 1				Session 2			
	Trial				Trial			
	1	2	3	4	1	2	3	4
I	HP	NE	NE	DN	DP	NE	NE	HN
II	HN	NE	NE	DP	DN	NE	NE	HP
III	DP	NE	NE	HN	HP	NE	NE	DN
IV	DN	NE	NE	HP	HN	NE	NE	DP

*HP* human positive, *HN* human negative, *DP* dog positive, *DN* dog negative, *NE* non-emotional

We applied different measures of precaution against habituation of the subjects: the first was to separate the experiment into two sessions (temporal distance was at least one day but no more than 4 weeks; mean = 18 days) to only play back four sounds per session. The second was to switch the rooms after each trial. The third was that the loudspeaker was never placed twice in the same location in one session. Thus, the situational context was always changed for each trial which should hinder quick habituation and, consequently, a reduction in the dogs’ response.

Before the experiment started, the dog was provided with 6 min in total to investigate both experimental rooms and to get accustomed to the situation. For this purpose, the dog, together with the owner and the experimenter, always entered first the experimental room in which the first trial was conducted later on through the respective front door. The connection door between both rooms was always closed. After 3 min—the dog was allowed to move freely in the room—the experimenter, together with the dog and the owner, went to the second experimental room through the connection door, which was again closed afterwards. Here the dog was again allowed to explore the room for 3 min. Then, the dog was taken on leash by the owner, and both entered the first experimental room again through the connection door and the first trial was conducted. The respective room for the first trial was counterbalanced between individuals and sessions. During the experiment, the experimenter was outside the testing room from where she observed the events in the room and started the different playbacks at the appropriate time.

For each trial, the owner entered the room through the connection door with the dog on leash and sat down on the chair. To avoid that the owners would react to the playbacks themselves, they wore wireless earphones during the experiment, through which they heard music. In addition, owners were asked to ignore their dogs and to read a magazine during the experiment. A trial always started with 5 min of accommodation in which the dog was held on a short leash by the owner. Following the accommodation period, the experimenter played an acoustic cue to the owner (via the earphones) as a signal to guide the dog onto the blanket where the dog should sit. This ensured that all subjects had one consistent starting point for each trial. The dog was unleashed, and a releasing command was given (to ensure that the dog connected this command correctly as being allowed to move, we checked it once together with the owner during the 6 min habituation period before the first trial started). Immediately afterwards, the experimenter started the playback of the stimulus and the dog was allowed to move freely in the room for 2 min. Thereafter, and upon a second acoustic cue to the owner, the dog was leashed again and led into the second testing room. The same procedure as in the first trial was repeated with a different stimulus. For the third trial, the owner led the dog into the first testing room again where the experimenter meanwhile had switched the position of the loudspeaker, and the procedure was repeated. Likewise, the forth trial was performed in the second room with the loudspeaker shifted to a different position. In the second session, the four remaining stimuli were presented following the same procedures.

### Analysis of the dogs’ behavioural response

All tests were video-taped with three cameras in each room, and subsequent analysis of the dogs’ behavioural response was made from these videos. For each trial, we analysed the subjects’ behavioural response based on continuous sampling during the playback (20 s) and 30 s after the end of the playback (altogether 50 s), using Solomon Coder (Solomon Coder, version 15.03.15, András Péter). The behavioural variables we considered most important for the research question were grouped into the following three behaviour categories:

1. The category “*Owner*-*oriented”* behaviour comprised two variables: “Look at owner” and “Approach the owner” (see Table 2). Both behaviour variables were not mutually exclusive as the dogs might have additionally expressed behaviours from the category “*Indicators for arousal and negative emotional states”.*

**Table 2:** List of the analysed variables of the behaviour category “*Owner*-*oriented”* and *“Loudspeaker*-*oriented”* with the respective definition and the type of recording

Category	Variable	Definition	Type of recording
1*. “Owner*-*oriented”*	“Look at owner”	Head orientation towards the owner	Duration
	“Approach the owner”	Dog was initiatively making direct body contact with the owner. The starting position in each trial was always the blanket next to the owner’s chair on which the dog was guided before the playback of each stimulus	Duration
2*. “Loudspeaker*-*oriented”*	“Look at loudspeaker”	Head orientation towards the predefined area of the loudspeaker which played back the stimulus	Duration
	“Approach the loudspeaker”	Approach of the loudspeaker, which played back the stimulus, to a distance of at least 10 cm. The starting position in each trial was always the blanket next to the owner’s chair on which the dog was guided before the playback of each stimulus	Duration
2. The category “*Loudspeaker*-*oriented”* behaviour comprised two variables: “Look at loudspeaker” and “Approach the loudspeaker” (see Table 2). Both behaviour variables were not mutually exclusive as the dogs might have additionally expressed behaviours from the category “*Indicators for arousal and negative emotional states”.*
3. The category “*Indicators for arousal and negative emotional states”* comprised ten behavioural variables that have been associated with arousal and negative emotional states such as distress in dogs in the literature (see Table 3). These behaviour variables were not mutually exclusive because the dogs might have additionally expressed other behaviours of the same category (except for the variable “Immobility/Freezing”, which was mutually exclusive for other behaviours of the same category) as well as of the category “*Owner*-*oriented”* or “*Loudspeaker*-*oriented”*.

**Table 3:** List of the analysed behaviours of the category “*Indicators for arousal and negative emotional states”* that were pooled for the variable “Relative Reactivity Score” with the definition, type of recording, and relative scientific literature that has previously associated the corresponding behaviours with arousal and negative emotional states in dogs

Variable	Definition	Type of recording	Relative scientific literature
“Barking”	Characteristic short loud vocalization	Frequency	e.g. Tod et al. 2005; Siniscalchi et al. 2008; Schilder and van der Borg 2004
“Whining”	Continuous high pitch vocalization	Frequency	e.g. Sheppard and Mills 2003; Siniscalchi et al. 2008; Quervel-Chaumette et al. 2016
“Yawning”	Opening of the mouth to the apparently fullest extent with eyes closing	Frequency	e.g. Beerda et al. 1998, 2000; Bellaio et al. 2009; Kuhne et al. 2014; c.f. Sonntag and Overall 2014; Quervel-Chaumette et al. 2016
“Scratching”	Scraping the own body with the claws of one hind leg	Frequency	e.g. Kuhne et al. 2014; Quervel-Chaumette et al. 2016
“Lip licking”	A part of the tongue is protruding and moved to upper lip	Frequency	e.g. Beerda et al. 1998; Bellaio et al. 2009; Rehn and Keeling 2011; c.f. Sonntag and Overall 2014; Quervel-Chaumette et al. 2016
“Shaking”	Rotating movements of the body	Frequency	e.g. Beerda et al. 1998, 2000; Siniscalchi et al. 2008; Bellaio et al. 2009; Pastore et al. 2011; Rehn and Keeling 2011; Kuhne et al. 2014; Quervel-Chaumette et al. 2016
“Stretching”	Stretching out either both hind legs or both forelegs away from the body	Frequency	e.g. Rehn and Keeling 2011; Kuhne et al. 2014
“Immobility/Freezing”	Immobile upright or sitting position with motionless head and tail for at least 1 s	Duration	e.g. King et al. 2003; Siniscalchi et al. 2008; c.f. Sonntag and Overall 2014; Travain et al. 2015; Kuhne 2016
“Tail wagging”	Repetitive, lateral wagging movements of the tail	Duration	e.g. Pastore et al. 2011; Rehn and Keeling 2011
“Panting”	Opened mouth while breathing short and quick	Duration	e.g. Sheppard and Mills 2003; Siniscalchi et al. 2008; Pastore et al. 2011; c.f. Sonntag and Overall 2014

We then pooled the ten behaviour variables of this category for the new variable “Relative Reactivity Score” (“RRS”), which represents the dogs’ overall behavioural response in terms of arousal and negative emotional valence. The behaviour variable “Immobility/Freezing” was additionally interpreted as a single, second variable in this behaviour category. Immobility, often referred to as freezing, is a bodily reaction of emotion (Spinka 2012) and, besides withdrawal, appeasement, and attack, a possible strategy to behave in negatively valenced situations (Marks 1987; Levine 1997; Kuhne et al. 2014). Therefore, we applied it as an additional indicator for a negative emotional state in the subjects.

The “RRS” is based on the methodology of previous studies that calculated behavioural scores to apply them as response and reactivity indices associated with negative emotional states in dogs (e.g. Branson and Rogers 2006; Siniscalchi et al. 2008). In both studies, a defined list of behaviours was analysed that were allocated a score of 1 each, when observed, respectively, and a score of 0 if not. The sum was used to calculate the overall reactivity index. However, in the context of the present study, we modified this methodology to not only indicate whether a behaviour was present in a trial or not, but also to incorporate the relative intensity of every behavioural variable in relation to its frequency or duration of expression. Thus, we calculated the “RRS” that included the ten behavioural variables of the category “*Indicators for arousal and negative emotional states”* for every subject in every trial. Hence, the “RRS” represents an overall score that, in line with the previous studies, comprises single scores of different behavioural variables. As in the mentioned studies, we allocated a single score 0, if a behaviour listed in the category “*Indicators for arousal and negative emotional states”* was not observed in a trial. However, if a listed behavioural variable was observed, we developed a method to allocate a scale of single scores ranging from 1 to 3 for this particular variable. The allocated single score was depending on the intensity (duration, respectively, frequency) of the expressed behaviour variable in the particular trial, in relation to the overall intensity of expression (duration, respectively, frequency) of this behavioural variable in all trials of all subjects. To obtain the thresholds for allocating the relative score (1–3) for each variable, we proceeded as follows:

1. For each of the ten behavioural variables of the category “*Indicators for arousal and negative emotional states”,* we excluded all zero values, representing those trials where the behaviour was not observed and therefore would become allocated the single score 0. The reason for this procedure was to extract only the values larger than zero, as only those were allocated a score ranging between 1 and 3.
2. For each of the ten behavioural variables of the category “*Indicators for arousal and negative emotional states”*, we computed tertiles from the remaining extracted values by dividing the respective data points into three equal subgroups. Consequently, each subgroup comprised a third of all values larger than zero.
3. The individual data point of each trial for each dog was then scored correspondingly to whether it ranged within the first tertile (=score 1), the second (=score 2) or whether it was larger than the second tertile (=score 3). This was possible for all but three variables (“Shaking”, “Stretching”, “Yawning”) where there was no large variance between individual data points, resulting in an equal first and second tertiles. In this case, when the data point ranged in the first and second tertiles, a score of 1 was given and a score of 2, when it exceeded the second tertile.
4. By summating all single scores of the ten behavioural variables together, we obtained the “RRS”. Overall, the lowest possible “RRS” was 0 and the highest possible score was 27.

The first author coded all videos, and a second researcher that was neither involved in planning nor in executing the study coded the videos of two trials of 30 dogs (altogether 60 trials) to determine interobserver reliability. The 60 trials analysed for interobserver reliability represent 14% of all trials of this study. We considered this amount as sufficient as the coded variables were always the same for every trial. The few disagreements of coding were solved in a joint decision among both coders and an external person not involved in planning or executing the study. Cohen’s Kappa, which was calculated to analyse variables that measured frequency of occurrence, showed results always above 0.72 with an overall mean agreement of 0.87. Cronbach’s Alpha, which was calculated to analyse variables that measured duration of occurrence, showed results always above 0.89 with an overall mean agreement of 0.95.

### Statistical analysis

Statistical analyses of the dogs’ responses were performed in RStudio 0.99.467. We used binomial models for binary response variables (GLMER, R-package “lme4” for logistic regression data, Bates et al. 2014) and general linear mixed effects models (GLMM, R-package “nlme”, Pinheiro et al. 2014) for all other response variables.

To analyse the “*Emotionality”* dimension, non-emotional trials were contrasted with pooled data of trials with emotional stimuli of both human and dog. To analyse the “*Species”* and the “*Valence”* dimension, trials with non-emotional stimuli were excluded from the dataset. These models included the species (dog, human) and the valence of the stimulus (positive, negative) as well as the interaction term between the two as predictors. Furthermore, we investigated whether the dogs’ responses differed, on the one hand, between non-emotional biotic and abiotic stimuli and, on the other hand, between the non-emotional human sound and the other non-emotional biotic stimuli.

All analyses were conducted with subject identity as a random factor and session (first, second) and subject’s sex, neuter status, and age as additional predictors, though the latter four were dropped from the models if the *p* value was above 0.1 and the model was calculated again. The residuals were assessed graphically and with Shapiro–Wilk’s test for normality (all *p* values > 0.05). When data were not normally distributed, suitable transformations were performed to approximate them to a normal distribution; appropriateness of these transformations was then determined by graphical assessment as well as Shapiro–Wilk’s test for normality. Transformations applied were mainly square root transformations (for the variables “RRS”, “Look at loudspeaker”, “Immobility/Freezing”). In case these transformations were not adequate to improve the data, either another exponent (2/5) was applied (for the variable “Look at owner”) or the variables were transformed into binary ones (for the variables “Approach the owner” and “Approach the loudspeaker”) and then analysed with binomial models.

## Results

### “*Emotionality”* dimension: comparing emotional with non-emotional sounds

The dogs’ behavioural responses differed between emotional and non-emotional sounds for nearly all analysed variables. For the behaviour category “*Owner*-*oriented”*, we observed a significantly shorter duration of “Look at owner” for trials with emotional sounds (Table 4). For the second variable of this behaviour category, “Approach the owner”, we did not find a significant effect based on the emotionality of the sounds but a session effect, as in the second session, the dogs were significantly more likely to approach their owner compared to the first session (Table 4). For the behaviour category “*Loudspeaker*-*oriented”*, we observed differences between trials with emotional and non-emotional sounds for both variables. Specifically, in trials with emotional sounds, the dogs looked significantly longer towards the loudspeaker area where the playback originated from (Table 4) and were more likely to approach the loudspeaker (Table 4). For the behaviour category “*Indicators for arousal and negative emotional states”*, we again observed differences between emotional and non-emotional sounds for both the “RRS” and “Immobility/Freezing”. Specifically, the calculated “RRS” was significantly higher in trials with emotional sounds, indicating that dogs increasingly expressed behaviours for arousal and negative emotional states in response to emotional sounds (Table 4). For this variable, we also found an effect of neuter status, as neutered dogs had a significantly lower score showing that they expressed indicators for arousal and negative emotional states less than intact dogs (Table 4). In addition, the dogs had a significantly lower score in the second session compared to the first (Table 4). Concerning the second variable of this behaviour category, “Immobility/Freezing”, the duration of how long the subjects remained immobile was significantly higher during emotional than non-emotional trials (Table 4). We also found an age effect as the older dogs were freezing less than the younger dogs (Table 4).

**Table 4:** Statistically significant results of the GLMM and GLMER analysis for the “*Emotionality”* dimension comparing trials with non-emotional stimuli to trials with emotional stimuli (pooled data of emotional dog and human sounds)

Behaviour category	Response variable	Predictor	Level	Statistic	χ^2^	Estimate	SE	*df*	Median	*P*
1. “*Owner*-*oriented*”	“Look at owner”	“*Emotionality”*	Emotional	*F* = 6.46		−0.23	0.09	365	0.02	0.012
	“Approach the owner”	Session	Two	*z* = 2.22	4.93	0.63	0.29	1	0.30	0.027
2. “*Loudspeaker*-*oriented”*	“Look at loudspeaker”	“*Emotionality”*	Emotional	*F* = 52.34		1.12	0.16	364	0.08	0.0001
	“Approach the loudspeaker”	“*Emotionality”*	Emotional	*z* = 2.68	7.15	0.92	0.35	1	0.32	0.008
3. *“Indicators for arousal and negative emotional states”*	“RRS”	“*Emotionality”*	Emotional	*F* = 5.14		0.14	0.06	364	0.06	0.024
		Neutered	Yes	*F* = 4.04		−0.34	0.17	51	0.06	0.050
		Session	Two	*F* = 6.15		−0.15	0.06	364	0.06	0.014
	“Immobility/Freezing”	“*Emotionality”*	Emotional	*F* = 34.41		0.94	0.16	365	0.01	0.0001
		Age		*F* = 8.92		−0.16	0.06	51	0.01	0.005

### “*Species”* dimension: comparing emotional dog with emotional human sounds

We did not find any significant differences comparing the behavioural reaction towards emotional dog and emotional human sounds (“Look at loudspeaker”: *F*_(1, 152)_ = 2.40, *p* = 0.124. All other variables *p* > 0.2), except for the variable “Immobility/Freezing” of the behaviour category “*Indicators for arousal and negative emotional valence”*. Specifically, the duration the dogs remained immobile was significantly increased in trials with dog compared to human sounds, independently of the valence of the sound (Table 5).

**Table 5:** Statistically significant results of the GLMM and GLMER analyses comparing trials with emotional dog sounds to trials with emotional human sounds (“*Species”* dimension) as well as comparing trials with negatively to trials with positively valenced sounds (“*Valence”* dimension)

Behaviour category	Response variable	Predictor	Level	Statistic	χ^2^	Estimate	SE	*df*	Median	*P*
1. “*Owner*-*oriented”*	“Look at owner”	Session	Two	*F* = 6.06		−0.29	0.12	152	0.04	0.015
	“Approach the owner”	Interaction effect: “*Species”* and “*Valence”*	Human (“*Species”*) and Positive (“*Valence”*)	*z* = 2.28	5.17	2.00	0.88	1	0.32	0.023
3. “*Indicators for arousal and negative emotional states”*	“RRS”	“*Valence”*	Positive	*F* = 5.57		−0.19	0.12	152	0.02	0.020
	“Immobility/Freezing”	“*Valence*”	Positive	*F* = 8.62		−0.41	0.31	152	0.05	0.004
		“*Species”*	Human	*F* = 4.67		−0.24	0.31	152	0.05	0.032

Furthermore, there was one significant interaction effect of the dimension “*Species”* with the dimension “*Valence”*; the dogs were more likely to approach their owners after hearing positively valenced human sounds (Table 5) (interaction effects for all other variables *p* > 0.15).

### “*Valence”* dimension: comparing positively with negatively valenced sounds

We found differences in the dogs’ response to positively and negatively valenced sounds of humans and conspecifics for both variables of the behaviour category “*Indicators for arousal and negative emotional valence”*. In trials with negative sounds, the “RRS” (Table 5) and the duration of how long the dogs remained immobile (Table 5) were significantly higher. Both effects were independent of the species from which the sound originated (interaction effects of “*Valence”* and “*Species”*: “RRS”: *F*_(1, 152)_ = 0.00, *p* = 1.0; “Immobility/Freezing”: *F*_(1, 152)_ = 1.05, *p* = 0.306). Furthermore, in the subset of data we used for analysing the “*Species”* and “*Valence”* dimensions, we found a significant effect of session for the variable “Look at owner”; in the second session, the dogs looked less long to their owners (Table 5).

### Additional analyses of non-emotional sounds

A difference in the dogs’ behavioural response to non-emotional biotic and abiotic stimuli was only observable for the variable “Approach the owner”; when hearing non-emotional biotic sounds, the dogs were less likely to approach their owners than when hearing non-emotional abiotic sounds (χ^2^_(1)_ = 6.84, *p* < 0.01). Concerning all other variables, there was no significant difference between non-emotional trials with biotic and abiotic sounds or between the non-emotional human sounds and the other non-emotional biotic sounds (*p* > 0.4).

## Discussion

In this playback study, we used different types of sounds to evaluate the influence of three dimensions on the dogs’ behavioural response with the aim to investigate emotional contagion in dogs. For reasonably interpreting the dogs’ behavioural response as being triggered by emotional contagion, three criteria have to be met: first, the behaviour should differ between emotional and non-emotional sounds (“*Emotionality”* dimension). Second, it should differ between positively and negatively valenced sounds (“*Valence”* dimension). And, third, to indicate emotional state-matching for negatively valenced states, behavioural indicators for negative emotions should increasingly be expressed in response to negatively compared to positively valenced sounds. Overall, the dogs’ behaviour response would have matched the valence of the perceived negative emotional sound.

Our results indicate that interpreting the dogs’ response to negative emotional sounds as being based on emotional contagion is reasonable. Five of six variables differed for the “*Emotionality”* dimension between non-emotional and emotional sounds (Table 4). When hearing the latter, dogs were more attentive to the loudspeaker of the playback and expressed more indicators for emotional states. One could argue that the dogs did not recognize the emotional features but that they attended more and responded stronger to sounds when they originated from humans and conspecifics (which was the case for all emotional sounds) as these sounds are probably more familiar and more salient. However, that the dogs’ response is indeed based on the emotional features and not solely on familiarity and salience is supported by the fact that the non-emotional biotic sounds also contained a human component, but dogs did not treat this sound differently than the other non-emotional biotic stimuli. Concerning the playback of dog sounds, it was not possible to apply a natural non-emotional vocalization comparable to the human sounds as it is assumed that dog vocalizations, such as barking, most probably convey information about the subject’s inner emotional state (Pongrácz et al. 2014). However, to confirm that it was indeed the emotional features of the dog sounds that triggered the subjects’ response and not solely familiarity or salience, future studies could also use non-vocal dog sounds (such as the sound of a dog walking or drinking) or, similarly as in research on avian vocalizations (see e.g. Araki et al. 2016), use scrambled dog vocalizations in addition to emotional sounds of conspecifics.

Concerning the species from which the emotional sound originated (“*Species”* dimension), we found a similar response after both human and conspecific stimuli for all but one of the variables, which was “Immobility/Freezing” (Table 5). The interruption or absence of ongoing movements is commonly termed freezing behaviour (e.g. Davis 1997; Levine 1997; Kuhne et al. 2014). As freezing is a behaviour response often expressed in social conflicting situations (Marks 1987), the increased freezing behaviour in response to dog stimuli, independent of their valence, might indicate that hearing unfamiliar dog sounds could generally cause higher social tension in the subjects compared to hearing unfamiliar human sounds. As familiarity seems to have an effect on empathetic responses of dogs to conspecifics (Quervel-Chaumette et al. 2016), it might be interesting to repeat the present study with positive and negative emotional sounds of familiar and unfamiliar dogs in order to compare whether the intensity of freezing is affected by the familiarity between both subjects.

Focussing on the “*Valence”* dimension, dogs differed in their behaviour response between positively and negative valenced sounds for the variables “RRS” and “Immobility/Freezing”, meaning they expressed significantly more indicators for arousal and negative emotional states after hearing negative emotional sounds (Table 5). Consequently, as the dogs’ responses matched the valence of the stimulus for the negatively valenced sounds, we consider this observation as an indicator for an emotional contagion response. Two previous studies that investigated emotional contagion in dogs analysed the emotional tone of the subjects’ behaviour in terms of emotional state postures (Custance and Mayer 2012; Yong and Ruffman 2014). Hence, they applied a rather qualitative behaviour analysis by coding emotional displays on the basis of the dogs’ overall body postures. In contrast, the present study applied a quantitative behaviour analysis by focussing on single predefined behaviours. Interpreting the dogs’ behaviour responses in terms of certain valenced states is generally a challenging task (Mendl et al. 2010). However, it is a prerequisite for labelling the behavioural response as being triggered by empathy (Edgar et al. 2012). The specific behaviour expressions analysed in the present study have already been shown to be related to arousal and distress states in dogs in previous studies (see references in Table 3). However, there are reasons to believe that these behavioural indicators for negative emotional states are context-specific and subjected to individual variability. Consequently, behaviour responses to stressful situations may differ between situations and individuals (Beerda et al. 1997, 1998). We accounted for this fact by including not only a few but ten different behaviour variables in the “RRS” and, thereby, were able to assess the intensity of arousal and negative emotional states despite interindividual differences.

This study was the first to contrast dogs’ behavioural responses to negative and positive emotions when investigating canine empathy. Traditionally, empirical research on this phenomenon has primarily focussed on negative emotional states (Edgar et al. 2012). This is also true for studies on canine empathy (e.g. Custance and Mayer 2012; Yong and Ruffman 2014; Quervel-Chaumette et al. 2016). From an evolutionary point of view, emotional states are considered to have adaptive value (Boissy et al. 2007) and so it is plausible that the contagious effect of negative emotions, which indicate aversive or dangerous situations, affect others’ behavioural responses more than positive ones (Preston and de Waal 2002). Accordingly, it is assumed that the functions of empathetic processes probably vary between positive and negative emotional situations (Edgar et al. 2012). Here, we demonstrated that the variables indicating arousal and negative emotional states were indeed significantly increased when hearing negatively valenced sounds but not positively valenced ones, despite equal degrees of attentiveness. Still, further attempts to determine valid and reliable behavioural indicators for positive emotional states in dogs are required as this is the basis to advance our understanding of emotional contagion and empathic-like behaviour for positive emotions in this species and, thereby, gaining a more comprehensive view on canine empathy. Furthermore, and not the least, this knowledge would be of high value for improving dog welfare and human–dog relationships.

## Appendix

See Table 6.

**Table 6:** Demographic data of participating dogs

Subject no.	Dog breed	S	N	*A*	Subject no.	Dog breed	S	N	*A*
1	Chinese Crested	F	N	2	28	Berner Sennenhund	M	Y	7.5
2	Chinese Crested	F	Y	12	29	Border Collie	F	Y	12.5
3	Mongrel	M	Y	4	30	Staffordshire Terrier	F	Y	4.5
4	Mongrel	M	Y	6	31	Labrador Retriever	M	Y	3
5	Mongrel	M	Y	2.5	32	Bearded Collie	M	N	6
6	Staffordshire Mongrel	F	Y	6	33	Australian Shepherd Mongrel	F	Y	8
7	Mongrel	F	Y	2.5	34	Border collie Mongrel	F	Y	3
8	Husky Mongrel	F	N	1.5	35	Australian Shepherd/Border Collie Mongrel	F	Y	4.5
9	Golden Retriever	F	Y	7	36	Bergspitz	F	N	1.5
10	Labrador Retriever	F	N	1	37	Dackel/Jack Russel/Pumi Mongrel	M	Y	1
11	Staffordshire Terrier^a^	M	Y	4	38	Balkanbracke Mongrel	F	Y	4
12	Akita Inu	F	Y	5.5	39	Chihuahua Mongrel	M	Y	10.5
13	Pinscher Mongrel	F	Y	9	40	Schnauzer Mongrel	M	N	5.5
14	Pinscher	F	Y	9	41	Chihuahua	F	Y	4.5
15	Zwergspitz	M	Y	11	42	Border Collie	M	N	2
16	Entlebucher Sennenhund	F	Y	3	43	Bearded Collie	M	N	2.5
17	Staffordshire Terrier	F	Y	3	44	Deutscher Boxer	F	N	3
18	Labrador Retriever Mongrel	F	Y	4.5	45	Zwergpinscher	F	N	3
19	Westhighland White Terrier	M	N	11	46	Border Collie	M	N	1.5
20	Magyar Viszlar	F	Y	4.5	47	Zwergpinscher Mongrel	F	Y	8
21	Tervueren	M	N	7.5	48	Golden Retriever	M	N	1.5
22	Airedale Terrier	M	N	8.5	49	Siberian Husky	M	N	3.5
23	Westhighland White Terrier^b^	F	Y	2	50	Siberian Husky	F	N	2
24	Chihuahua	F	Y	6	51	Standard Poodle	M	Y	2
25	Chihuahua Mongrel	F	Y	4.5	52	Standard Poodle/Herding Dog Mongrel	F	Y	3
26	Herding Dog Mongrel	F	N	4	53	Mongrel	F	Y	5
27	Rhodesian Ridgeback	F	Y	3.5					

^a^Heard one playback less

^b^Dropped out after 1st session

*S* sex, *N* neuter status, *A* age (years)
